# *Sp1*-Mediated circRNA *circHipk2* Regulates Myogenesis by Targeting Ribosomal Protein Rpl7

**DOI:** 10.3390/genes12050696

**Published:** 2021-05-08

**Authors:** Junyu Yan, Yalan Yang, Xinhao Fan, Yijie Tang, Zhonglin Tang

**Affiliations:** 1 Shenzhen Branch, Guangdong Laboratory for Lingnan Modern Agriculture, Agricultural Genomics Institute at Shenzhen, Chinese Academy of Agricultural Sciences, Shenzhen 518124, China; 82101181031@caas.cn (J.Y.); yangyalan@caas.cn (Y.Y.); fanxinhao@caas.cn (X.F.); tangyijie@caas.cn (Y.T.); 2 Genome Analysis Laboratory of the Ministry of Agriculture and Rural Affairs, Agricultural Genomics Institute at Shenzhen, Chinese Academy of Agricultural Sciences, Shenzhen 518124, China; 3 Research Centre of Animal Nutritional Genomics, State Key Laboratory of Animal Nutrition, Institute of Animal Sciences, Chinese Academy of Agricultural Sciences, Shenzhen 518124, China; 4 Kunpeng Institute of Modern Agriculture at Foshan, Foshan 528226, China; 5 GuangXi Engineering Centre for Resource Development of Bama Xiang Pig, Bama 547500, China

**Keywords:** circRNA, *circHipk2*, Rpl7, myogenesis, skeletal muscle, *Sp1*

## Abstract

Circular RNAs (circRNAs) represent a class of covalently closed single-stranded RNA molecules that are emerging as essential regulators of various biological processes. The circRNA *circHipk2* originates from exon 2 of the *Hipk2* gene in mice and was reported to be involved in acute promyelocytic leukemia and myocardial injury. However, the functions and mechanisms of *circHipk2* in myogenesis are largely unknown. Here, to deepen our knowledge about the role of *circHipk2*, we studied the expression and function of *circHipk2* during skeletal myogenesis. We found that *circHipk2* was mostly distributed in the cytoplasm, and dynamically and differentially expressed in various myogenesis systems in vitro and in vivo. Functionally, overexpression of *circHipk2* inhibited myoblast proliferation and promoted myotube formation in C2C12 cells, whereas the opposite effects were observed after *circHipk2* knockdown. Mechanistically, *circHipk2* could directly bind to ribosomal protein Rpl7, an essential 60S preribosomal assembly factor, to inhibit ribosome translation. In addition, we verified that transcription factor *Sp1* directly bound to the promoter of *circHipk2* and affected the expression of *Hipk2* and *circHipk2* in C2C12 myoblasts. Collectively, these findings identify *circHipk2* as a candidate circRNA regulating ribosome biogenesis and myogenesis proliferation and differentiation.

## 1. Introduction

Myogenesis plays important roles in skeletal muscle regeneration and growth [1,2]. It is a multistep process, including myoblast proliferation, myocyte differentiation, fusion of multinuclear myotubes with the central nucleus, and further muscle fiber maturation [3,4]. It is well known that myogenesis is regulated by myogenic regulatory factors (*MRFs*), such as the muscle bHLH proteins *Myf5*, *myogenin* (*MyoG*), *MyoD* and *MRF4*, and the *MEF2* family members [2,5,6]. Recently, increasing evidence suggested that other RNA types, including microRNAs (miRNAs), long non-coding RNAs (lncRNAs), and circular RNAs (circRNAs), play important roles during myogenesis in mammals [7,8]. For example, *miR-1* and *miR-133* are well-known myomiRs, *miR-1* promotes myogenesis by targeting histone deacetylase 4 (*HDAC4*), and *miR-133* enhances myoblast proliferation by repressing serum response factor (*SRF*) [9]. LncRNA *linc-RAM* regulates the expression of myogenic genes by binding to *MyoD*, thus enhancing myogenic differentiation [10]. However, compared to miRNAs and lncRNAs, studies of circRNAs in myogenesis are still limited.

As a novel type of ncRNA derived from exons, introns, or intergenic regions, circRNAs have a covalently closed continuous loop, are generally expressed at low levels, and often exhibit cell type-specific and tissue-specific patterns [11,12,13]. Recently, many studies have revealed the crucial functions of circRNAs in myogenesis as a miRNA sponge [14,15]. For example, *circLMO7* regulates myoblast differentiation and survival by sponging *miR-378a-3p* [15]. *circRILPL1* acts as a *miR-145* sponge to facilitate the proliferation and differentiation of myoblasts via the *IGF1R*/*PI3K*/*AKT* signaling pathway [16]. In addition, *circZNF609* and *circFAM188B* can be combined with polysomes for translation, thereby regulating myoblast proliferation [17,18]. However, little circRNAs functioning as protein sponges have been discovered in myogenesis, and need to be systematically explored. 

The circular RNA *circHipk2* is reported to be a regulator of many cellular processes, such as proliferation, apoptosis, and autophagy [19,20,21]. The host gene of *circHipk2*, serine/threonine kinase homeodomain-interacting protein kinase 2 (*Hipk2*), is widely involved in multiple biological processes, including cell proliferation, cell differentiation, and apoptosis [22,23,24]. In myoblasts, the *Hipk2* gene is involved in cell cycle regulation, and functions alternatively as a corepressor that inhibits myocyte enhancer factor 2 (*MEF2*)-dependent gene expression [25,26]. In our previous work, we found that *circHipk2* was upregulated in differentiated myotubes compared to proliferating myoblasts in C2C12 myoblast cells. However, the function and mechanism of *circHipk2* in myogenesis are still unknown. In this study, we profiled the temporal expression patterns of *circHipk2* in vitro and in vivo, and analyzed the function and mechanism of *circHipk2* in myogenesis. The results revealed that *circHipk2* inhibited myoblast proliferation and promoted differentiation by targeting ribosomal protein L7 (Rpl7), a ribosomal protein that is a component of the 60S subunit. Our recent study suggested that overexpression of transcription factor *Sp1* promoted differentiation and repressed proliferation in C2C12 myoblasts [27]. In this study, we verified that *Sp1* could directly bind to the promoter of *circHipk2* and thus affect its transcription activity. Our findings indicated that *circHipk2* may exert regulatory functions in skeletal muscle development.

## 2. Materials and Methods

### 2.1. Cell Isolation and Culture

HEK293T and C2C12 cells were cultured in Gibco Dulbecco’s modified Eagle medium (DMEM) (Gibco, Co Dublin, Ireland) supplemented with 10% FBS in a humidified incubator with 5% CO_2_ at 37 °C. To induce myogenic differentiation, C2C12 cells were incubated in DMEM supplemented with 2% heat-inactivated horse serum (Gibco).

### 2.2. Plasmid Construction and RNA Interference

The promoter of *Hipk2* was subcloned into the pGL3-Basic vector (Promega, Madison, WI, USA). To construct the overexpression vectors, the full-length sequence of mouse *circHipk2* and *Sp1* were cloned into the pLC5-circ vector and pcDNA3.1 vector, respectively (Geenseed Biotech, Guangzhou, China). The siRNAs of these genes were synthesized in Ribobio Biotech (Guangzhou, China), and their sequences are listed in Table S1.

### 2.3. circRNA Pull-Down Assay

A pLC5-*circHipk2*-Flag vector and pLC5-*circHipk2* vector were constructed by Geenseed Biotech (Guangzhou, China). Cellular lysates from C2C12 myoblasts with a transfecting pLC5-*circHipk2*-Flag vector or pLC5-*circHipk2* were divided into an input group and pull-down group, in which the pull-down group was used for *circHipk2* pull-down, and the protein level of RpL7 was normalized to GAPDH (38 kDa) in the input group. Briefly, streptavidin beads were prewashed and blocked according to the manufacturer’s instructions. Cellular lysates (pull-down group) were incubated with 100 μL streptavidin beads overnight at 4 °C. *circHipk2*-Flag-bound proteins were eluted with 100 μL urea buffer (2 M urea and 50 mM Tris (pH 7.5)) supplemented with 1 mM dithiothreitol and 5 μg/μL trypsin and LysC. After alkylation with 5 mM iodoacetic acid (IAA), proteins were proteolytically digested with trypsin and LysC for 24 h. Peptides were acidified, loaded on SDB-RPS material and eluted and dried. Peptides were resuspended in 2% acetonitrile (ACN) and 0.1% trifluoroacetic acid (TFA), then MS analyses were performed.

### 2.4. Cell Proliferation Assay

Cell proliferation was determined by a 5′ethynyl-2′-deoxyuridine (EdU) incorporation assay kit (Ribobio) and Cell Counting Kit-8 (CCK-8) reagent (Dojindo). The C2C12 cells were cultured in GM containing EdU for 2 h. Ten microliters of CCK-8 were added to each well of a 96-well plate containing cells and incubated at 37 °C for 45 min.

### 2.5. RNA Preparation, RT-PCR, and RT-qPCR

Total RNA was extracted from skeletal muscles and cells using TRIzol reagent (Invitrogen, Carlsbad, CA, USA) according to the manufacturer’s instructions. For RNase R treatment, total RNA (2 μg) was cultivated for 30 min at 37 °C with or without 3 U/mg of RNase R (Epicentre). RNAs from the nucleus and cytoplasm of C2C12 myoblasts were separated using a Cytoplasmic and Nuclear RNA Purification Kit (Norgen, Thorold, ON, Canada) following the manufacturer’s instructions. cDNAs were prepared using reverse transcriptase (Thermo Fisher Scientific, Waltham, MA, USA). Oligo(dT) primers were used for coding genes and random primers were used for circRNAs. Reverse transcription PCR (RT-PCR) analysis was performed to detect the existence of *circHipk2* in cDNAs and genomic DNAs using divergent primers by KOD-Plus-Neo (Toyobo, Osaka, Japan). The following three-step protocol was used: one cycle at 94 °C for 2 min, followed by 34 cycles at 98 °C for 10 s, 60 °C for 30 s, and 68 °C for 30 s. Finally, 25 µL of the total PCR volume were used according to the manual protocol. Analysis of gene expression was performed with SYBR Green Master Mix (ABI) by quantitative real-time PCR (RT-qPCR). RT-qPCR data were analyzed using the ΔΔCt method as in our previous report [27]. The primer sequences used in the present study are listed in Table S1.

### 2.6. Western Blotting

The total proteins from C2C12 cells were lysed in RIPA lysis buffer supplemented with a protease inhibitor cocktail (Roche, Mannheim, Germany). The membranes were blocked with 5% BSA for 1.5 h at room temperature, and subsequently probed with primary antibodies overnight at 4 °C. The following dilutions were used for each antibody: myogenin (1:1000; Proteintech, Rosemont, IL, USA), MyHC1 (1:1000; Dshb, New Delhim, India), GAPDH (1:1000; Proteintech), PCNA (1:1000; Proteintech), cyclin E1 (1:1000; Proteintech), and Rpl7 (1:1000; Abcam, Cambridge, MA, USA). The membranes were then washed with PBS-Tween and incubated for 30 min with horseradish peroxidase-conjugated secondary antibodies (Proteintech). Protein bands were detected after treatment with SuperSignal West Femto agent (Thermo Scientific).

### 2.7. RNA Fluorescence In Situ Hybridization Assay (RNA-FISH)

The RNA-FISH assay was performed in C2C12 myoblasts following the manufacturer’s instructions (GEFAN). The probe sequence for *circHipk2* is 5′-CGGTAGTATCTGGATTGCAAGTACGTAGAGCAGACAGCTTTGGAC-3′, and that for *Rpl7* is 5′-TCCTTGCCTTTCGAAGTGTCTTCAGGGCAAACTTCTTCCTCAGGC-3′. Briefly, cells were seeded onto a cover-glass in 6-well plates, cultured to 70–80% confluence, and fixed. Following treatment with 0.1% Triton X-100, cells were incubated with 20 mg/mL probes overnight at 37 °C. Nuclei was counterstained with DAPI. Images were acquired using an FV1200 laser confocal microscope (Olympus, Tokyo, Japan).

### 2.8. Immunostaining Staining

C2C12 myoblasts were fixed on coverslips with 4% paraformaldehyde for 10 min, washed with PBS, and treated with 0.3% Triton X-100/PBS at room temperature for a further 20 min. Cells were rinsed with PBS twice and blocked in 5% goat serum including Tris-buffered saline buffer for one hour at room temperature, followed by incubation with primary antibodies for 2 h. Sections or cells were then washed in PBS and incubated with secondary antibodies for 1h. Primary and secondary antibodies were anti-MyHC1 (1:100, DSHB) and Alexa Fluor 594 goat anti-mouse IgG (1:400, Proteintech), respectively.

### 2.9. RNA Immunoprecipitation (RIP)

A Magna RIP RNA-Binding Protein Immunoprecipitation Kit (BersinBio) was used to determine the interaction between *circHipk2* and Rpl7. Antibodies used for the RIP assay included anti-Rpl7 and control IgG (Millipore, Billerica, MA, USA). The RNA/protein complex was recovered using protein G Dynabeads™ and washed with RIPA buffer several times. Following digestion with proteinase K, RNA was recovered using TRIzol and analyzed by RT-qPCR.

### 2.10. Chromatin Immunoprecipitation (ChIP)

A ChIP assay was performed using a ChIP kit (EMD Millipore Corporation, Billerica, MA, USA) following the manufacturer’s instructions as previously described [27]. Briefly, the crosslinking reaction was terminated by glycine in *Sp1* overexpression C2C12 cells treated with 1% formaldehyde. Next, samples were lysed for 10 min in lysis buffer on ice, and sonicated to an average length of 200–1000 bp. The anti-Rpl7 antibody (Abcam) was added to form the antibody-target protein–DNA complex, and protein A–Sepharose beads were used to immunoprecipitate the complex. After washing and reversing the crosslinks, precipitated DNA was amplified by RT-PCR. Primer sequences are provided in Table S1.

### 2.11. Statistical Analysis

Statistical analysis was performed using the SPSS 13.0 software package. Each data value represents the mean± S.D./S.E.M. for 3–5 separate experiments. The significance (such as *p* < 0.05) of differences between the experimental variables was determined using Welch’s *t*-test.

## 3. Results

### 3.1. circHipk2 Is a Candidate Regulator of Myogenesis

**Figure 1:**
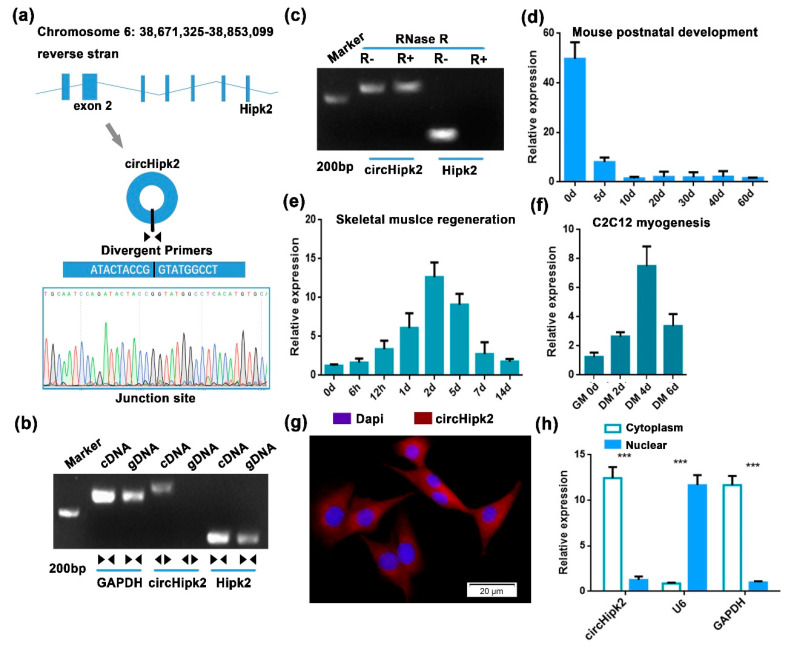
*circHipk2* is a candidate regulator of myogenesis and skeletal muscle regeneration. (**a**) Schematic illustration of *circHipk2* formation via the circularization of exon 2 in *Hipk2* gene. The back-splice junction sequences and RT-PCR product of *circHipk2* were validated by Sanger sequencing. (**b**) RT-PCR was performed to detect the existence of *circHipk2* and *Hipk2* from cDNA and gDNA in C2C12 myoblasts using the divergent and convergent primers. (**c**) RT-PCR was conducted to determine *circHipk2* in C2C12 myoblasts treated with RNase R. (**d**–**f**) RT-qPCR analysis of the expression of *circHipk2* during postnatal development in the hind leg muscles of C57BL/6 mice (**d**), during CTX-induced TA muscle regeneration (**e**) and during C2C12 myogenesis (**f**). (**g**) RNA-FISH was performed to determine *circHipk2* subcellular localization in C2C12 myoblasts. Blue indicates nuclei stained with DAPI; red indicates the RNA probe that recognizes *circHipk2*. The scale is 20 μm. (**h**) Verification of *circHipk2* localization by subcellular fractionation. The error bars depict the mean ± S.D. of samples from 3 individuals. *** *p* < 0.001.

In our previous study, several differentially expressed circRNAs were identified by microarray analysis during myoblast differentiation. Among them, *circHipk2* caught our attention, since *circHipk2* was more highly expressed in differentiated myotubes compared to proliferating myoblasts in C2C12 cells, and its host gene *Hipk2* was reported to be time-dependently expressed in skeletal muscle [28]. Sequence analysis suggested that *circHipk2* was a single exonic circRNA generated by exon 2 of the linear *Hipk2* sequence in mice. The junction sequence of *circHipk2* was confirmed by divergent primers, then validated by Sanger sequencing (Figure 1a). RT-PCR results showed that *circHipk2* was only detected in cDNA, thus ruling out the existence of *circHipk2* in genomic DNA (gDNA), whereas the convergent primers amplified *Hipk2* from both cDNA and gDNA (Figure 1b). In addition, we found that *circHipk2* was resistant to RNase R digestion, whereas the linear *Hipk2* transcript was digested by RNase R (Figure 1c).

We next examined the temporal expression patterns of *circHipk2* in vitro and in vivo. The age-dependent decrease in the expression of *circHipk2* was found in the mouse hind leg muscles during postnatal development (Figure 1d). Using the cardiotoxin (CTX)-induced skeletal muscle damage and regeneration model, we analyzed the expression of *circHipk2* during skeletal muscle regeneration and found that *circHipk2* was highly expressed 2 d post injury and subsequently decreased in expression thereafter (Figure 1e). Further investigation confirmed that *circHipk2* was expressed at low levels in the proliferating myoblasts but at high levels during differentiation (Figure 1f). Expression patterns of *circHipk2* were different from that of the linear *Hipk2* transcript, indicating that *circHipk2* might function independently of *Hipk2* (Figure S1). Then, we determined the subcellular localization of *circHipk2*. Based on RNA-FISH (Figure 1g) and chromatin fractionation (Figure 1h), we found that *circHipk2* was mostly distributed in the cytoplasm, suggesting that *circHipk2* might play a role in post-transcriptional regulation. Taken together, these data indicate that *circHipk2* potentially is involved in myogenesis and skeletal muscle development.

### 3.2. circHipk2 Represses Myoblast Proliferation but Promotes Differentiation

**Figure 2:**
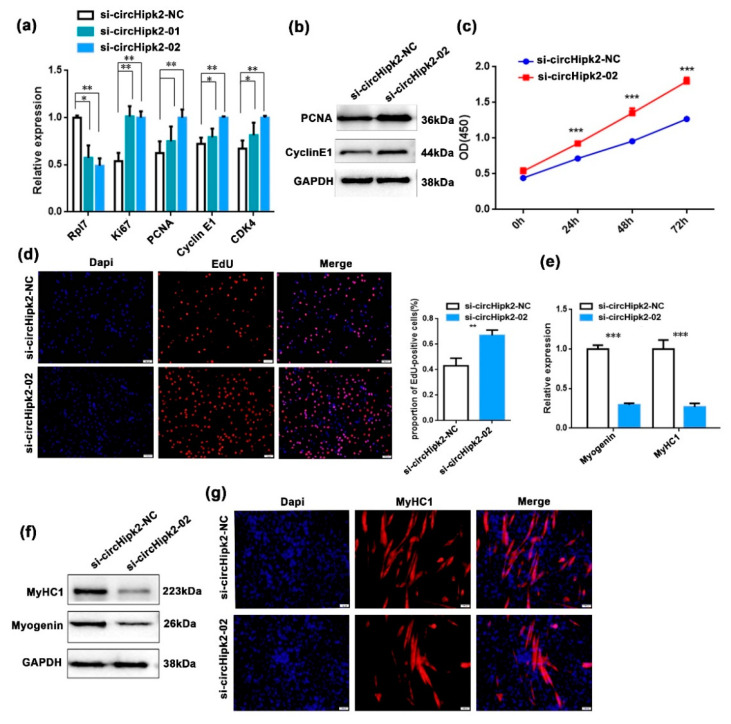
*circHipk2* represses C2C12 myoblast proliferation but promotes differentiation. (**a**,**b**) The expression of proliferation and cell cycle markers was quantitated by RT-qPCR (**a**) and Western blotting (**b**) in C2C12 myoblasts. Data are presented as the mean ± S.D. *N* = 3 per group. * *p* < 0.05, ** *p* < 0.01 and *** *p* < 0.001. (**c**) Cell proliferation was assessed using the CCK-8 assay after transfection with si-*circHipk2*-02 or si-*circHipk2*-NC. (**d**) EdU assay to assess cell proliferation after transfection with si-*circHipk2*-02 or si-*circHipk2*-NC in C2C12 myoblasts. Cell proliferation indices were assessed after treatment with EdU and counted using ImageJ. EdU staining (red) for positive cells; DAPI staining (blue) for cell nuclei. The scale bars represent 100 μm. (**e**,**f**) The expression levels of *myogenin* and MyHC1 were detected by RT-qPCR (**e**) and Western blotting (**f**) after transfection with si-*circHipk2*-02 or si-*circHipk2*-NC in C2C12 myoblasts. (**g**) Immunofluorescence analysis of MyHC1 cells (red) after transfection with si-*circHipk2*-02 or si-*circHipk2*-NC in C2C12 myoblasts. The scale bars represent 100 μm.

To explore the function of *circHipk2* in myogenesis, we first investigated whether *circHipk2* regulated C2C12 myoblast proliferation. We constructed a *circHipk2* overexpression (*circHipk2*-OV) vector (Figure S2A) and designed two small interfering RNAs (si-*circHipk2*-01 and si-*circHipk2*-02) to target the back-splicing junction of *circHipk2*. After transfecting them into C2C12 myoblasts, the si-*circHipk2*-02 fragment showed higher silencing efficiency than the si-*circHipk2*-01 fragment, and was chosen for subsequent analysis (Figure 2a). We observed that knockdown of *circHipk2* by si-*circHipk2*-02 significantly upregulated the expression of proliferation markers (*Ki67*, *PCNA*, *cyclin E1*, and *CDK4*) at both mRNA (Figure 2a) and protein levels (Figure 2b), whereas overexpression of *circHipk2* significantly decreased the expression of these genes (Figure S2B). Based on the EdU incorporation assay and CCK-8 assay, we observed that knockdown of *circHipk2* significantly increased cell proliferation activities in C2C12 myoblasts (Figure 2c,d). In contrast, overexpression of *circHipk2* caused the opposite effects (Figure S2B,C).

Next, we investigated the role of *circHipk2* on myoblast differentiation, and found *circHipk2* prevented the expression of the myoblast determination factors (*myogenin* and *MyHC1*) at both mRNA (Figure 2e) and protein levels (Figure 2f), whereas overexpression of *circHipk2* increased the expression of these differentiation markers (Figure S2D). The immunofluorescence assay suggested that knockdown of *circHipk2* dramatically blocked myotube formation when compared with the control group (Figure 2g), whereas overexpression of *circHipk2* promoted myotube formation (Figure S2E). Collectively, these results indicated that *circHipk2* repressed myoblast proliferation and promoted differentiation in C2C12 cells.

### 3.3. circHipk2 Directly Binds to Ribosomal Protein Rpl7

**Figure 3:**
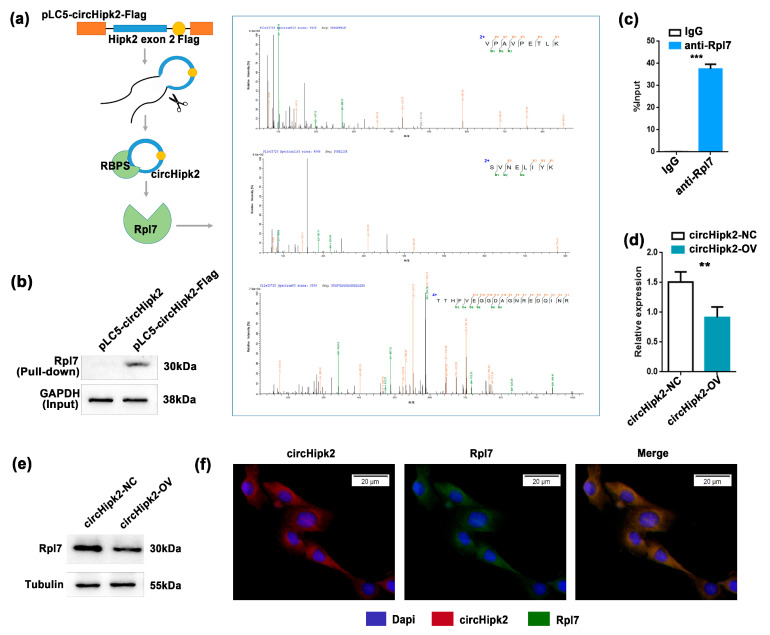
*circHipk2* functions by binding to ribosomal protein *Rpl7*. (**a**) Schematic of λN-peptide-mediated capture of pLC5-*circHipk2*-Flag from cellular lysates of overexpressing C2C12 myoblasts, and label-free mass spectrometric quantification. Experiments were performed in a pool of three biological replicates (quadruplicate measurements). (**b**) Western blotting of *Rpl7* after λN-peptide-mediated pLC5-*circHipk2*-Flag capture in C2C12 myoblasts. (**c**) Fold enrichment of *circHipk2* quantitated by RT-qPCR after the RIP assay with the *Rpl7* antibody. IgG was used as the negative control. (**d**,**e**) The expression of *Rpl7* was detected by RT-qPCR (**d**) and Western blotting (**e**) after transfection with *circHipk2*-OV and their negative controls in C2C12 myoblasts. The error bars depict the mean ± S.D. of samples from 3 measurements. ** *p* < 0.01 and *** *p* < 0.001. (**f**) RNA-FISH assay was performed to determine *Rpl7* and *circHipk2* subcellular localization. Scale bar, 20 μm.

To identify the downstream targets of *circHipk2* in myogenesis, we next performed a proteomic screen to identify potential *circHipk2*-binding proteins (Figure 3a). We generated C2C12 myoblasts with stable overexpression of pLC5-*circHipk2*-Flag engineered to contain RNA hairpin BoxB sequences (Figure 3a), this allowed the capture of *circHipk2*-binding proteins in cellular lysates via high-affinity interaction of the BoxB RNA hairpin with bacteriophage λ transcriptional antiterminator protein N (λN-peptide) coupled to beads [29,30]. Then, we used 2 μg of precipitated protein for label-free mass spectrometric analyses. A total of 49 proteins were identified to be significantly enriched in extracts from pLC5-*circHipk2*-Flag C2C12 myoblasts (Table S2). Gene ontology (GO) and KEGG pathway enrichment analysis revealed that the majority of target proteins were involved in ribosome biogenesis and assembly (28%) (Figure S3A,B), suggesting that *circHipk2* may play a crucial role in these important cellular processes.

Strong binding was determined for ribosomal protein L7 (*Rpl7*) (Figure 3a), and we further validated the interaction between *circHipk2* and *Rpl7* by pull-down and RNA immunoprecipitation (RIP) assays. As expected, λN-peptide-mediated pull-down of pLC5-*circHipk2*-Flag followed by Western blotting validated that *circHipk2* directly bound to *Rpl7* protein (Figure 3b). Meanwhile, we found a significant enrichment of *circHipk2* in the *Rpl7* pull-down samples compared to the IgG negative controls (Figure 3c). In addition, overexpression of *circHipk2* could significantly reduce the expression of *Rpl7* at both mRNA (Figure 3d) and protein levels (Figure 3e). Then, we determined the subcellular localization of *Rpl7* in C2C12 myoblasts. RNA-FISH showed that *Rpl7* was mainly distributed in the cytoplasm, the same as *circHipk2* (Figure 3f). Overall, these results indicated that *circHipk2* directly bound to Rpl7 and inhibited its biogenesis.

### 3.4. The Function Role of Rpl7 in Myogenesis Proliferation and Differentiation

**Figure 4:**
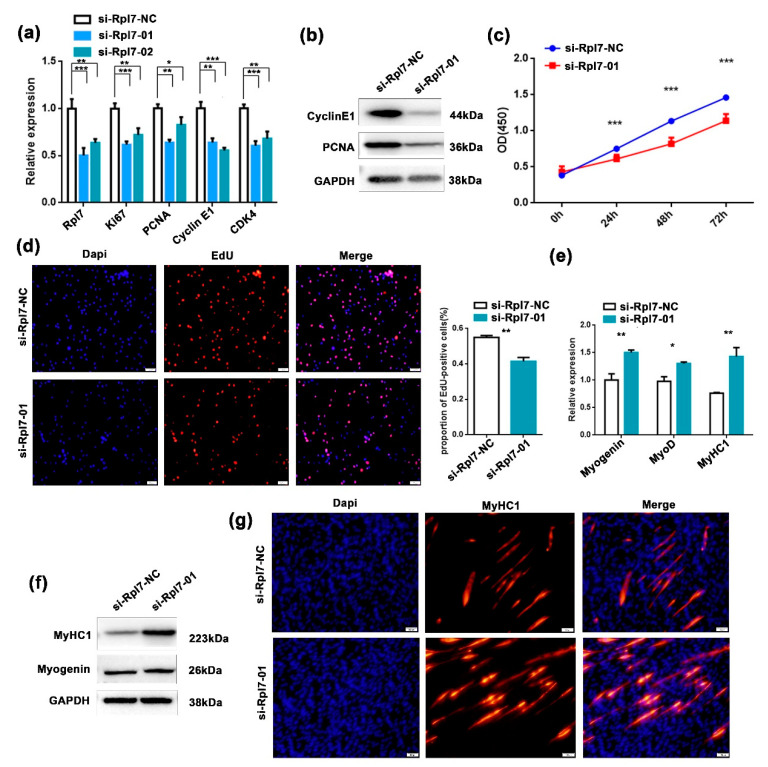
The role of *Rpl7* in skeletal muscle proliferation and differentiation. (**a**,**b**) The expression of proliferation and cell cycle markers was quantitated by RT-qPCR (**a**) and Western blotting (**b**) in C2C12 myoblasts after transfection with si-*Rpl7* or its control. Data are presented as the mean ± S.D. *N* = 3 per group. * *p* < 0.05, ** *p* < 0.01 and *** *p* < 0.001. (**c**) Cell proliferation was assessed using the CCK-8 assay after transfection with si-Rpl7-01 or its control. (**d**) EdU assay to assess cell proliferation after transfection with si-*Rpl7*-01 or si-*Rpl7*-NC in C2C12 myoblasts. Cell proliferation indices were assessed after treatment with EdU and counted using ImageJ. EdU staining (red) for positive cells; DAPI staining (blue) for cell nuclei. The scale bars represent 100 μm. (**e**,**f**) The mRNA and protein expression levels of myogenic differentiation markers *myogenin* and *MyHC1* were detected by RT-qPCR (**e**) and Western blotting (**f**) after transfection with si-*Rpl7*-01 or si-*Rpl7*-NC in C2C12 myoblasts. (**g**) Immunofluorescence analysis of MyHC1 cells (red) after transfection with si-*Rpl7*-01 or its control in C2C12 myoblasts. The scale bars represent 100 μm.

Previous studies suggested that *Rpl7* could affect trophoblast differentiation, and is abnormally expressed in colon cancer and other diseases [31,32]. However, the functions of *Rpl7* in myogenesis have not been reported. To investigate the role of *Rpl7* in myoblast proliferation, we designed two siRNAs targeting *Rpl7* (si-*Rpl7*-01 and si-*Rpl7*-02). After evaluating their silencing efficiencies, the siRNA si-*Rpl7*-01 was chosen for the next analysis. The expression of *Ki67*, *PCNA*, *cyclin E1*, and *CDK4* were analyzed by RT-qPCR and Western blotting after transfection with si-*Rpl7*-01 in C2C12 cells. We found that the expression of these genes was significantly upregulated after *Rpl7* knockdown (Figure 4a,b). Based on the EdU incorporation and CCK-8 assays, we observed that knockdown of *Rpl7* significantly increased the activity of cell proliferation (Figure 4c,d).

Next, we investigated the role of *Rpl7* on myoblast differentiation, and found that *Rpl7* knockdown significantly upregulated the expression of differentiation markers (*myogenin* and *MyHC1*) at both mRNA (Figure 4e) and protein (Figure 4f) levels. As shown in Figure 4g, the knockdown of *Rpl7* dramatically accelerated myotube formation when compared with the control group by immunostaining of *MyHC1*. Taken together, these data indicated that *Rpl7* promoted proliferation but had inhibitory effects on myoblast differentiation.

### 3.5. Sp1 Modulates the Transcription of circHipk2

**Figure 5:**
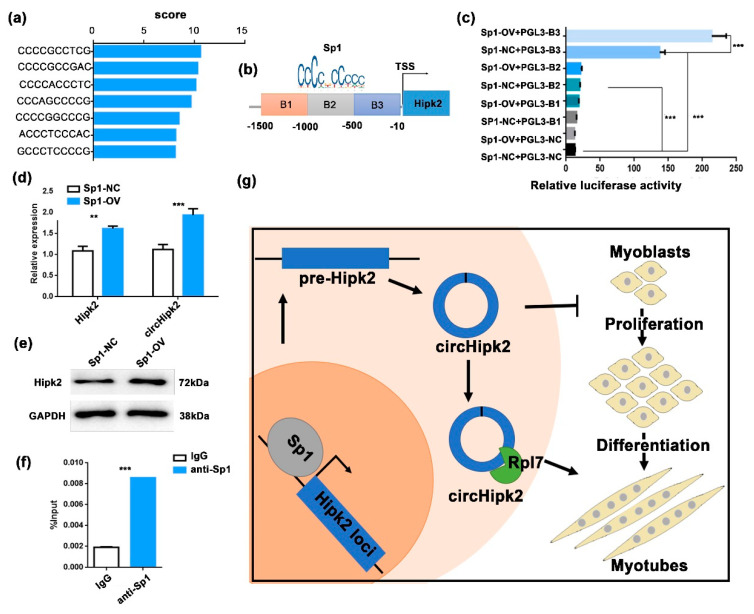
*Sp1* modulates the transcription of *circHipk2*. (**a**) The score of enriched TF-binding motif *Sp1* in the *Hipk2* promoter. (**b**) Schematic illustration of putative binding regions of *Sp1* in the *Hipk2* promoter. (**c**) The relative luciferase activities were detected in HEK293T cells co-transfected with luciferase reporter plasmids containing putative *Sp1*-binding sites in the promoter sequence and overexpression plasmids of *Sp1*. (**d**,**e**) The expression levels of *Hipk2* and *circHipk2* were detected in C2C12 myoblasts after transfecting with *Sp1*-OV or *Sp1*-NC by RT-qPCR (**d**) and Western blotting (**e**). The error bars depict the mean ± S.D of three replicates. ** *p* < 0. 01, *** *p* < 0.001. (**f**) The enrichment of *Sp1* binding at *Hipk2* promoter was detected by ChIP–qPCR in C2C12 myoblasts. *IgG* was used as a negative control. (**g**) A model of *circHipk2* functions and regulation mechanism in myogenesis.

To explore the mechanism that mediates the biogenesis of *circHipk2* in myoblasts, we predicted the TFs that could potentially bind to the *Hipk2* promoter via JASPAR (http://jaspar.genereg.net, accessed on 8 August 2020). More than seven putative *Sp1* binding sites in the promoter of *Hipk2* were predicted (Figure 5a). *Sp1* is a well-known activator of *MyoD* and a suppressor of *CDKN1A*. It plays an important role in muscle cell proliferation and differentiation [27,33,34]. We cloned three continuous regions containing these binding sites (B1–B3) and constructed a series of luciferase reporter vectors (Figure 5b). The luciferase reporter assays showed that *Sp1* significantly promoted the luciferase activity of the B3 promoter (Figure 5c). Meanwhile, overexpression of *Sp1* significantly upregulated the expression of linear *circHipk2* and *Hipk2* (Figure 5d,e), implying that *Sp1* may regulate the expression of *Hipk2* and *circHipk2*. Moreover, chromatin immunoprecipitation (ChIP)–qPCR analysis demonstrated that *Sp1* could directly bind to the B3 of the *Hipk2* promoter (Figure 5f). Therefore, these data illustrate that *Sp1* is an upstream regulator of *Hipk2* and directly affects the expression of *Hipk2* and *circHipk2* in myoblasts.

## 4. Discussion

It is well known that the multistep process of myogenesis is sophisticatedly controlled by the expression of myogenic genes, such as myogenic regulatory factors (*MRFs*) and paired box (*PAX*) genes [1,35,36]. However, mounting studies suggest that myogenesis is also regulated by a variety of certain RNA types, including miRNAs, lncRNAs, and circRNAs [7,37]. In this study, we found that the circular RNA *circHipk2* was significantly upregulated in C2C12 myoblast differentiation and mouse skeletal muscle regeneration, indicating that it had a potential effect in regulating skeletal muscle development. Further investigation demonstrated the anti-proliferation and pro-differentiation function of *circHipk2* in myoblasts by acting as an *Rpl7* protein sponge. The results highlight the functions and mechanism of *circHipk2* in myogenesis.

The aberrant expression of circRNAs is also reported to be associated with muscular disease [7,12,13]. However, only a few of circRNAs have been functionally and mechanistically characterized in skeletal muscle. For instance, *circFUT10* reduces proliferation and facilitates differentiation of myoblasts by sponging *miR-133a* [38]. *circFGFR4* can regulate myogenesis by sponging *miR-107* [39]. *circTTN* acts as a sponge of *miR-432* to facilitate the proliferation and differentiation of myoblasts via the *IGF2*/*PI3K*/*AKT* signaling pathway [40]. Our microarray studies suggested that *circHipk2* was differentially expressed during myoblast differentiation in C2C12 cells. Here, we demonstrated the function of *circHipk2*, repressing myoblast proliferation but promoting differentiation. These results uncover *circHipk2* as a new regulator of skeletal muscle development. Autophagy and apoptosis also play important roles in myogenesis, upregulation of apoptosis increases autophagy, and autophagy promotes myoblasts catabolism, supporting the normal differentiation of myoblasts [41,42]. In a future study, the function of *circHipk2* in autophagy and apoptosis should also be explored. 

circRNAs modulate gene expression by various mechanisms, including functioning as miRNA sponges, RNA-binding protein (RBP) sponges, transcription regulators, and templates for translating peptides [37,43,44]. *ciRS-7* (also known as *CDR1as*), the first miRNA sponge to be identified, is well known for sponging *miR-7* [45]. *ci-ankrd52* is directly associated with the chromatinized DNA at its host locus and stimulates the expression of the host genes from which it derives [46]. *circ-ZNF609* is known to be associated with heavy polysomes, and produces a detectable endogenous protein in murine and human myoblasts [17,47]. circRNAs directly interact with many different RBPs to act as protein sponges [48,49]. *circMbl* harbors a binding site for *Mbl* and *MBNL1*, to which the binding of *Mbl* (or *MBNL1*) facilitates the looping of the nascent RNA to promote *circMbl* biogenesis, and an autoregulatory circuit may exist [50]. *circPABPN1* suppresses the translation of nuclear poly(A) binding protein 1 (PABPN1) by sequestering the RBP Hu-antigen R (HUR) [51]. However, the expression, function, and mechanism of circRNAs functioning as a protein sponge in myogenesis are largely unknown. *Rpl7* is a coregulator of several nuclear receptors, which regulates protein expression in mammalian cells by tunable synthetic translational inhibition [31,32,52]. When functioning as a ribosomal protein, *Rpl7* forms homodimers which interact with specific sites on poly(A) RNA and DNA and associates with the large ribosomal subunit as part of the translational machinery [52,53]. In this study, we identified *circHipk2* acting as a binding protein sponge in myogenesis. *circHipk2* inhibited myoblast proliferation and promotes differentiation by binding ribosomal protein *Rpl7*. Function studies suggested that *Rpl7* promoted myoblast proliferation and inhibited differentiation by affecting ribosomal translation in all probability. Meanwhile, with a combination of bioinformatic tools [54,55,56,57], we predicted that *circHipk2* contained an ORF coding 359aa, probably contained six high-confidence m^6^A sites, and potential IRES sequences. Further studies are needed to explore the protein-coding potential of *circHipk2*.

The expression of circRNAs is also regulated by upstream transcription factors. It is reported that transcription factor *Twist1* promoted *circ-Cul2* transcription through binding to its promoter [58]. *E2F1* and *EIF4A3* could increase the expression level of *circSEPT9* [59]. *Sp1* is a zinc finger transcription factor that binds GC-rich DNA motifs to regulate thousands of genes, and is involved in many critical cellular functions, such as cell growth, differentiation, and apoptosis [60,61]. Recent studies suggested that *Sp1* could function as a DNA methylation-related modulator and an activator of myogenic differentiation, and plays a critical role in skeletal muscle development [27,33]. In this study, we verified that *Sp1* directly binds to the promoter of *circHipk2* and promoted the expression of both *Hipk2* and *circHipk2* in C2C12 myoblasts. In addition to transcription factors, competing RNA pairings modulate the transcription of circRNAs. At the individual gene level, alternative circularization suggests that one gene locus can produce multiple circRNAs with mechanisms related to alternative back-splicing and alternative splicing site selection. For example, both the *CAMSAP1* and *CRKL* locus could produce two major circRNA isoforms [62,63]. The circRNA microarray profiling suggested that the *Hipk2* locus could produce an exonic circRNA and an intronic circRNA. However, detailed mechanisms in the regulatory process need to be further elucidated.

## 5. Conclusions

In summary, our data highlight that the *Sp1*-mediated *circHipk2*-*Rpl7* axis regulates myogenesis (Figure 5g). Our study suggests that *circHipk2* is a critical myogenesis regulator during skeletal muscle development, and provides a new insight to understand the mechanism of circRNAs in myogenesis.

## Data Availability

Not applicable.

## Supplementary Materials

The following are available online at https://www.mdpi.com/article/10.3390/genes12050696/s1, Figure S1. The temporal expression patterns of *Hipk2*. Figure S2. Overexpression of *circHipk2* prevents C2C12 myoblast proliferation but promotes differentiation. Figure S3. Identification of *circHipk2*-binding proteins. Table S1. Information regarding the primers and siRNAs used in the present study. Table S2. Summary of pLC5-*circHipk2*-Flag binding proteins.
