# Supplementary material for: Sp1-Mediated circRNA circHipk2 Regulates Myogenesis by Targeting Ribosomal Protein Rpl7
Source: Genes (Basel). 2021 May 8;12(5):696. doi: 10.3390/genes12050696 (PMC8151578; doi:10.3390/genes12050696)
Supplement: Supplementary file 1 [file genes-12-00696-s001.zip › Supplementary/Figure supplement1-3.pdf]

Figure supplement 1-3

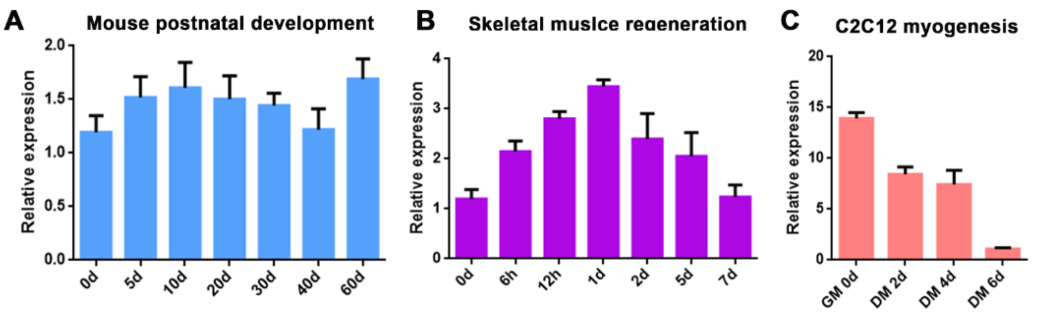

Figure S1. The temporal expression patterns of Hipk2. The expression of Hipk2 (**A**) during postnatal development in the hind leg muscles of C57BL/6 mice, (**B**) during CTX-induced TA muscle regeneration and (**C**) in C2C12 myogenesis.

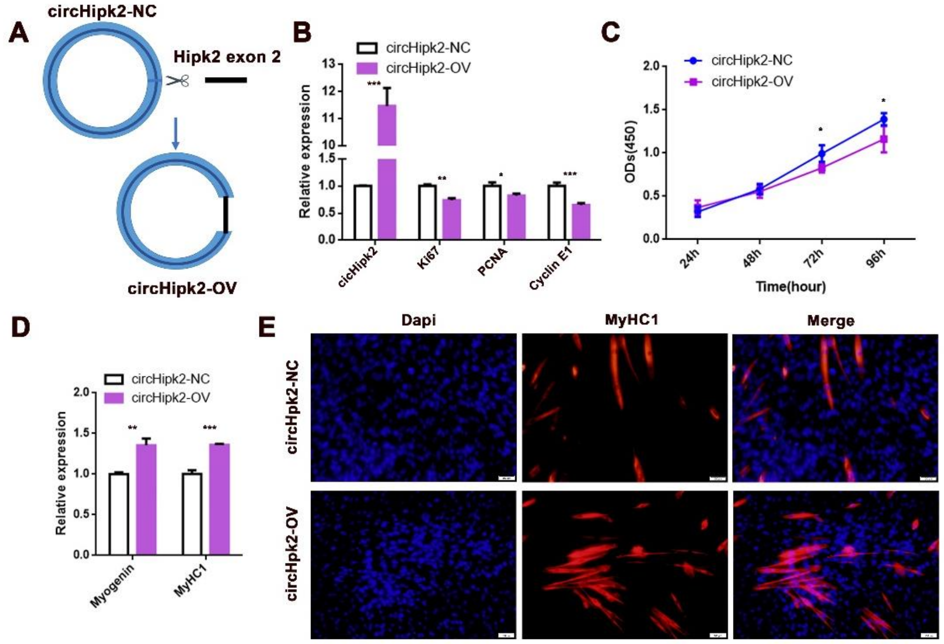

Figure S2. Overexpression of circHipk2 prevents C2C12 myoblasts proliferation but promotes differentiation. (**A**)Schematic diagram of the efficiency of circHipk2 overexpression vector construction. (**B**) The expression of proliferation and cell cycle markers was quantitated by RT-qPCR in C2C12 myoblasts after transfection with circHipk2-OV or circHipk2-NC. (**C**) Cell proliferation was assessed using the CCK-8 assay after transfection with circHipk2-OV or circHipk2-NC. (**D**) The expression of myogenic differentiation markers was quantitated by RT-qPCR in C2C12 myoblasts after transfection with circHipk2-OV or circHipk2-NC. Data are presented as the mean ±S.D. N=3 per group. * $P < 0.05$ and ** $P < 0.01$. (**E**) Immunofluorescence analysis of MyHC1 cells (red) after transfection with circHipk2-OV or circHipk2-NC in C2C12 myoblasts.; the scale bars represent 100 μm.

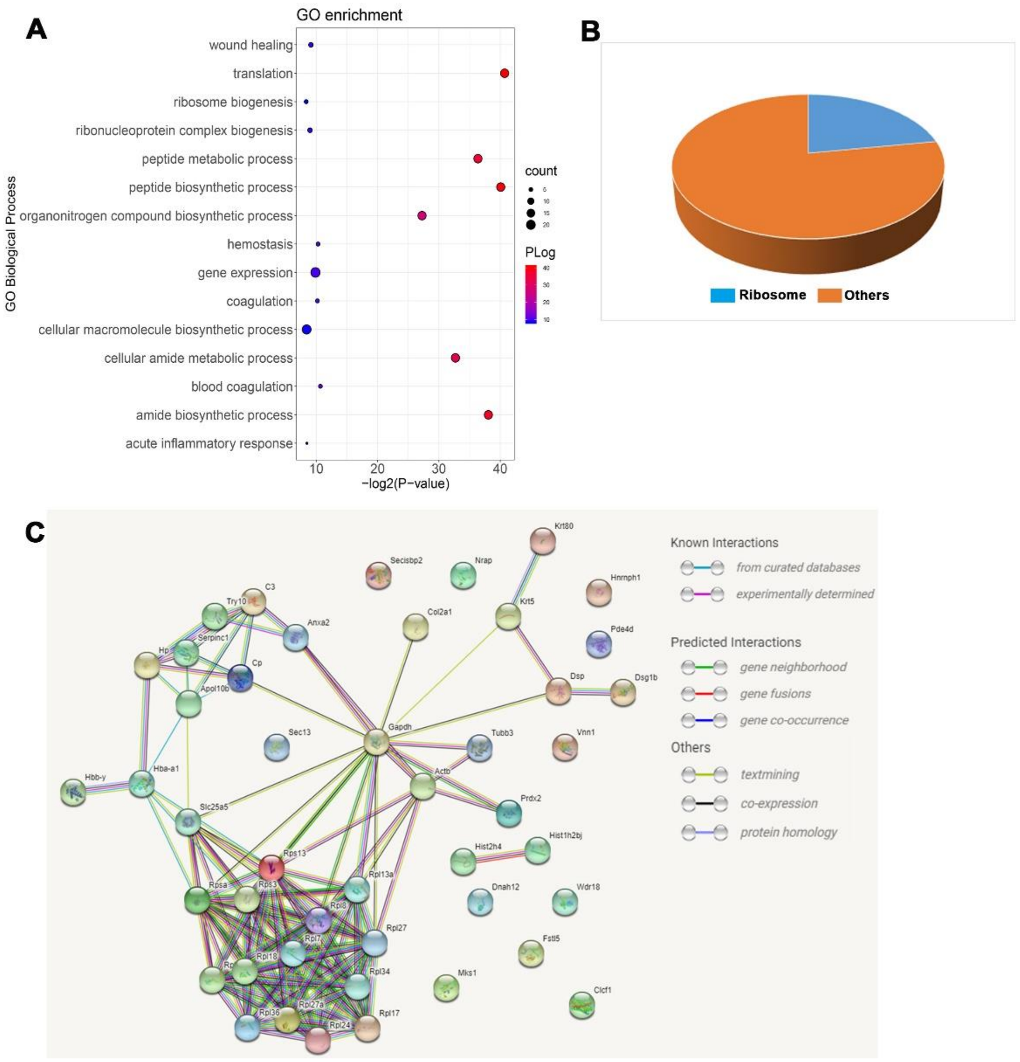

Figure S3. Identification of circHipk2 binding proteins. (**A**) GO analyses of cirHipk2 pull down proteins. (**B**) Summary of circHipk2 binding proteins according to KEGG pathway analysis and published functions. (**C**)The interaction between circHipk2 binding proteins.
